# Evidence of mutant huntingtin and tau-related pathology within neuronal grafts in Huntington’s disease cases

**DOI:** 10.1016/j.nbd.2024.106542

**Published:** 2024-05-27

**Authors:** Shireen Salem, Mitchell D. Kilgore, Mehwish Anwer, Alexander Maxan, Dan Child, Thomas D. Bird, C. Dirk Keene, Francesca Cicchetti, Caitlin Latimer

**Affiliations:** a Centre de Recherche du CHU de Québec, Axe Neurosciences, T2-07, 2705, Boulevard Laurier, Québec, QC G1V 4G2, Canada; b Departement de Médecine Moléculaire, Université Laval, Québec, QC, Canada; c Departement de Psychiatrie et Neurosciences, Université Laval, Québec, QC, Canada; d Department of Laboratory Medicine and Pathology, Neuropathology Division, University of Washington, Seattle, WA, USA; e Department of Neurology, University of Washington, Seattle, WA, USA; f Geriatric Research, Education, and Clinical Center (GRECC), VA Puget Sound Health Care System, Seattle, WA, USA

**Keywords:** Huntington’s disease, Fetal neural transplantation, Mutant huntingtin protein, tau

## Abstract

A number of post-mortem studies conducted in transplanted Huntington’s disease (HD) patients from various trials have reported the presence of pathological and misfolded proteins, in particular mutant huntingtin (mHtt) and phosphorylated tau neuropil threads, in the healthy grafted tissue. Here, we extended these observations with histological analysis of post-mortem tissue from three additional HD patients who had received similar striatal allografts from the fetal tissue transplantation trial conducted in Los Angeles in 1998. Immunohistochemical staining was performed using anti-mHtt antibodies, EM48 and MW7, as well as anti-hyperphosphorylated tau antibodies, AT8 and CP13. Immunofluorescence was used to assess the colocalization of EM48^+^ mHtt aggregates with the neuronal marker MAP2 and/or the extracellular matrix protein phosphacan in both the host and grafts. We confirmed the presence of mHtt aggregates within grafts of all three cases as well as tau neuropil threads in the grafts of two of the three transplanted HD patients. Phosphorylated tau was also variably expressed in the host cerebral cortex of all three subjects. While mHtt inclusions were present within neurons (immunofluorescence co-localization of MAP2 and EM48) as well as within the extracellular matrix of the host (immunofluorescence co-localization of phosphacan and EM48), their localization was limited to the extracellular matrix in the grafted tissue. This study corroborates previous findings that both mHtt and tau pathology can be found in the host and grafts of HD patients years post-grafting.

## 1. Background

Huntington’s disease (HD) is a fatal, autosomal dominant neurodegenerative disease caused by a CAG expansion in exon 1 of the huntingtin gene (*Htt*) which is located on the short arm of chromosome 4. Affected individuals typically harbor over 39 CAG repeats and present with significant and increasingly debilitating changes in behavior, cognition, and motor function between the ages of 35 and 50 (Huntington, 1872; Walker, 2007). The CAG repeats generate a polyglutamine expansion leading to the production of a misfolded form of the Htt protein – referred to as mutant huntingtin (mHtt) – which in turn aggregates within cells and ultimately causes dysfunction and death. While several brain regions are affected in HD, most notable is the marked atrophy of the caudate nucleus and putamen of which the degeneration of GABAergic medium spiny neurons is the substrate (Vonsattel and DiFiglia, 1998).

Available treatments for HD are limited and provide no significant long-term benefits (Ajitkumar and De Jesus, 2023). For this reason, several experimental therapeutic strategies have been attempted, including transplantation of striatal neurons. In the 1990s, a number of open label trials were conducted in small cohorts of patients who received either whole fetal tissue or cell suspension striatal implants (Sramka et al., 1992; Madrazo et al., 1993; Gaura et al., 2004; Furtado et al., 2005; Bachoud-Lévi et al., 2006; Gallina et al., 2008a; Gallina et al., 2008b; Reuter et al., 2008; Cicchetti et al., 2009; Gallina et al., 2010; Barker et al., 2013; Paganini et al., 2014; Madrazo et al., 1995; Bachoud-Lévi, 2020; Philpott et al., 1997; Kopyov et al., 1998; Freeman et al., 2000a; Bachoud-Lévi et al., 2000a; Bachoud-Lévi et al., 2000b; Hauser et al., 2002; Rosser et al., 2002), but both failed to generate measurable improvements in clinical presentation. While evidence from multiple post-mortem histological evaluations suggests that the viability of the transplanted tissue/cells is fairly robust, the overall integration of this tissue within the host remains limited (Freeman et al., 2000a; Capetian et al., 2009; Cisbani and Cicchetti, 2014). Further, long-surviving allografts are characterized by signs of degeneration similar to those found in the surrounding diseased host parenchyma (Cicchetti et al., 2009; Freeman et al., 2000a; Keene et al., 2009; Cisbani et al., 2013; Cicchetti et al., 2014; Cisbani et al., 2017; Maxan and Cicchetti, 2018) including the presence of Htt-immunopositive aggregates and tau-positive neurofibrillary tangles, neuropil threads, and inclusions (Cicchetti et al., 2014; Cisbani et al., 2017).

This report focuses on the post-mortem histological findings from an individual with HD who received a fetal graft as part of a transplant trial conducted at Good Samaritan Hospital in Los Angeles, California in 1995 (case 1). We also review the histological observations from two additional cases that were previously reported (case 2; referred to as patient 2 in (Keene et al., 2007), and case 3; described in (Keene et al., 2009)). Additionally, we specifically analyzed all three cases for the presence of mHtt and hyperphosphorylated tau proteins in the grafted tissue.

## 2. Methods

### 2.1. Patient selection

The neural transplantation trial performed in the three HD subjects reported here has been described previously (Kopyov et al., 1998; Keene et al., 2009; Keene et al., 2007). Briefly, all protocols were approved by the Institutional Review Board at Good Samaritan Hospital in Los Angeles, CA. HD patients were selected, with appropriate consent, based on criteria proposed in the Core Assessment Program for Intracerebral Transplantation for Huntington’s disease (Quinn et al., 1996). Inclusion and exclusion criteria included chorea as the primary clinical symptom, radiographically confirmed striatal atrophy, family history of HD, positron emission tomography (PET) scan-confirmed striatal hypometabolism, and no serious complicating medical or psychiatric conditions (Kopyov et al., 1998). Patients underwent neuropsychological and Unified Huntington’s Disease Rating Scale (UHDRS) prior to transplantation and post-operatively for two years.

### 2.2. Transplantation

The recommendations established by the National Institutes of Health at the time of this trial were followed for procurement of donor fetal tissues as described in detail previously (Kopyov et al., 1998). Preoperative planning including graft selection and transplant coordinates were determined immediately prior to surgery using brain magnetic resonance imaging (MRI). Bilateral craniotomies were performed, and a single piece of fetal tissue (80–120 μL) was stereotactically injected at the appropriate coordinates.

### 2.3. Brain collection and tissue preparation

Informed consent for research brain donation was obtained from the legal next of kin according to protocols approved by the University of Washington Institutional Review Board that conform to the provisions of the Declaration of Helsinki and preserve patient anonymity. The brains of the transplanted patients were removed within 48 hours (hrs) of death, fixed by immersion in 10% neutral buffered formalin, after which the brainstem and cerebellum were dissected from the cerebrum via axial transection through the midbrain. The cerebral hemispheres were then sectioned coronally at 0.5–1 cm thickness while the brainstem was sectioned axially and the cerebellum sagitally, both at ~0.5 cm thickness. A microtome was used to cut 4 μm thick tissue sections from formalin-fixed paraffin embedded tissue blocks.

### 2.4. Immunohistochemical evaluation

For case 1 we performed immunohistochemistry for MAP2 (mouse monoclonal; MAB341, Millipore; 1:500) on tissue sections of the striatum. This was conducted using diamino-benzidine tetrachloride as the chromogen substrate according to published protocols (Keene et al., 2009; Keene et al., 2007), and allowed us to evaluate the general health of the grafts and delineate the anatomical borders with the host. MAP2 evaluation has been previously reported on cases 2 and 3 (see (Keene et al., 2009; Keene et al., 2007) for details).

All cases were also assessed for the presence of mHtt aggregates and tau pathology within the grafted tissue. Double immunohistochemical staining was performed using the anti-mHtt antibody EM48 (mouse anti-human huntingtin clone EM48, MAB5374, Millipore; 1:500) or MW7 (mouse anti-human; obtained from the Developmental Studies Hybridoma Bank; 1:100) in combination with the anti-tau antibodies AT8 (mouse anti-human phosphorylated tau Ser202 and Thr205, MN1020, Thermo Fisher Scientific; 1:500) or CP13 (mouse anti-human phosphorylated tau Ser202; 1:500, a generous gift from the late Dr. Peter Davies, Feinstein Institute for Medical Research, NY, USA) (Cisbani and Cicchetti, 2014; Cicchetti et al., 2014; Roberson et al., 2007). Sections were de-paraffinized at 60°C for 20 minutes (min), cleaned in citrosolv (22–143–975, Thermo Fisher Scientific) for 10 min and hydrated in descending grades of ethanol (100%, 95%, 70% and 50%). After briefly rinsing with water, antigen retrieval was performed by incubating sections in 10 mM sodium citrate (W302600, Sigma-Aldrich) buffer (pH 6.2) for 30 min at 95°C. After a cool down at room temperature (RT), sections were washed for 5 min with 0.2M potassium phosphate buffer saline (KPBS) thrice. Endogenous peroxidase activity was quenched by adding a 3% aqueous H_2_O_2_ solution to the sections which were then treated with a blocking solution (5% normal goat serum (053–150, Wisent Bioproducts) and 0.1% Triton X-100 (T8787, Sigma-Aldrich) 10% in KPBS) for 45 min and subsequently incubated with an anti-mHtt antibody (EM48 or MW7, in blocking solution) overnight at 4°C. Following KPBS washes, goat anti-mouse biotinylated secondary antibody (BA-9200, Vector Laboratories) was added for 1 hr at RT. Sections were ultimately washed in KPBS buffer thrice and placed in a solution containing avidin-biotin peroxidase complex (PK-6100, ABC Elite kit, Vector Laboratories) for 1 hr at RT. Following two 10-min washes with 0.2M acetate imidazole solution (pH 7.2), sections were treated with a Ni-DAB solution (3,3-diaminobenzidine tetrahydrochloride (D5905, Sigma-Aldrich), ammonium nickel sulphate (A1827, Sigma-Aldrich), 1M sodium acetate (pH 7.2; S2889, Sigma-Aldrich), 0.2M imidazole (O3196, Thermo Fisher Scientific) and 0.1% hydrogen peroxide (516813, Sigma-Aldrich) at RT. The chromogenic reaction with Ni-DAB gave a purple-black color to mHtt aggregates. The sections were then thoroughly washed in KPBS (3 X 10 min), blocked for 45 min, followed by an overnight incubation with the second primary antibody (AT8 or CP13) solution at 4°C. After washing with KPBS, sections were incubated in goat anti-mouse biotinylated secondary antibody and then in ABC Elite kit for 1 hr each. The staining was concluded by treating the sections with a DAB solution (3,3-diaminobenzidine tetrahydrochloride in Tris-imidazole 0.2M pH 7.2) followed by washing with KPBS. Sections were dehydrated, coverslipped with DPX (13512, Electron Microscopy Science) and viewed under a brightfield microscope. To ensure accurate identification of mHtt aggregates, which, given their size, could be mistaken as artefacts, sections were first viewed at 60X and the presence of aggregates confirmed at 100X magnification.

All antibodies required initial antigen retrieval with incubation in boiling citrate buffer at pH 6.2. Sections of cerebral cortex was included as a positive control for all antibodies. Negative controls were also run and consisted of secondary antibodies of the appropriate species in the absence of primary antibody.

### 2.5. Immunofluorescence

Immunofluorescence was performed to further assess the localization of mHtt aggregates within neurons and/or the extracellular matrix. Sections were de-paraffinized and re-hydrated as for immunohistochemistry staining. They were then washed (3 X 8 min) in 1x phosphate buffer saline (PBS) (BP399–20, Fisher Bioreagent) containing 0.1% Tween 20 (also known as polysorbate 20) (PBST) (BP337–500, Fisher Bioreagent) and incubated for 5 min in 1X of the antigen retrieval solution QuatroSol II Tris EDTA Buffer (K086-RUO, Diagnostic BioSystems) at RT prior to a 40 min incubation in this solution at 95°C in a water bath. Slides were cooled down for 20 min at RT, washed in PBST (2 X 8 min) and blocked for 1 hr at RT in 10% donkey serum (D9663, Sigma-Aldrich), 1% Albumin Bovine Serum (BSA) (ALB001, BioShop), and 1% Triton X-100 in 1x PBS. They were then incubated overnight at 4°C in the following primary antibodies: mouse anti-mHtt (MAB5374, Millipore; 1:200), rabbit anti-MAP2 (17490–1-AP, Protein tech, 1:500) and rat anti-phosphacan hPTPβ/3 (extracellular matrix) (MAB2688, R&D Systems, 1:100). The next day, slides were washed (3 X 8 min) in PBST and incubated for 1 hr in secondary antibodies diluted in 1% BSA and 1% Triton X-100 in 1x PBS: Alexa 488 donkey anti-mouse (A2122, Invitrogen; 1:500), Alexa 647 donkey anti-rabbit (A31573, Invitrogen; 1:500) and Alexa 594 donkey anti-rat (A21209, Invitrogen; 1:250). After 3 X 5 min washes in PBST, slides were incubated with 0.022% 4’6-diamidino-2-phenylindole (DAPI) nuclear stain (D3571, Invitrogen) for 10 min diluted in PBST. Sections were washed again (3 X 5 min) in PBST and finally incubated with 0.1% Sudan Black B (199664, Sigma) diluted in 70% ethanol for 30 min at RT. Four 5 min washes in PBST were conducted and slides were then left to dry prior to being coverslipped with Fluoromount G mounting medium (00495802, Thermo Fisher Scientific).

### 2.6. Image acquisition

Immunohistochemistry photomicrographs were taken using a Lumina HR Camera (MicroBrightField Bioscience) attached to an E800 Nikon microscope (Nikon Instruments, Canada) and Stereo investigator software. Fluorescent photomicrographs were procured using a Zeiss Imager Z.2 AXI0 upright confocal microscope and Zeiss Zen Imaging software. Images were prepared using Fiji and panels were assembled using Adobe Illustrator CS5.

### 2.7. Assessment of tau pathology and mHtt aggregate number and size

Observations included location of staining (brain area, graft area) and types of tau patterns (neurofibrillary tangles, neuropil threads and inclusions). The density of EM48^+^ mHtt deposits, reported as the number of aggregates/mm^2^ of tissue, was assessed in the cortex and putamen of the host brain as well as of the grafted tissue using standard techniques (Cisbani et al., 2013). For aggregate size, the perimeter of sampled aggregates was delineated and assessed in the above-mentioned structures. All three cases were plotted individually.

## 3. Results

**Fig. 1.**
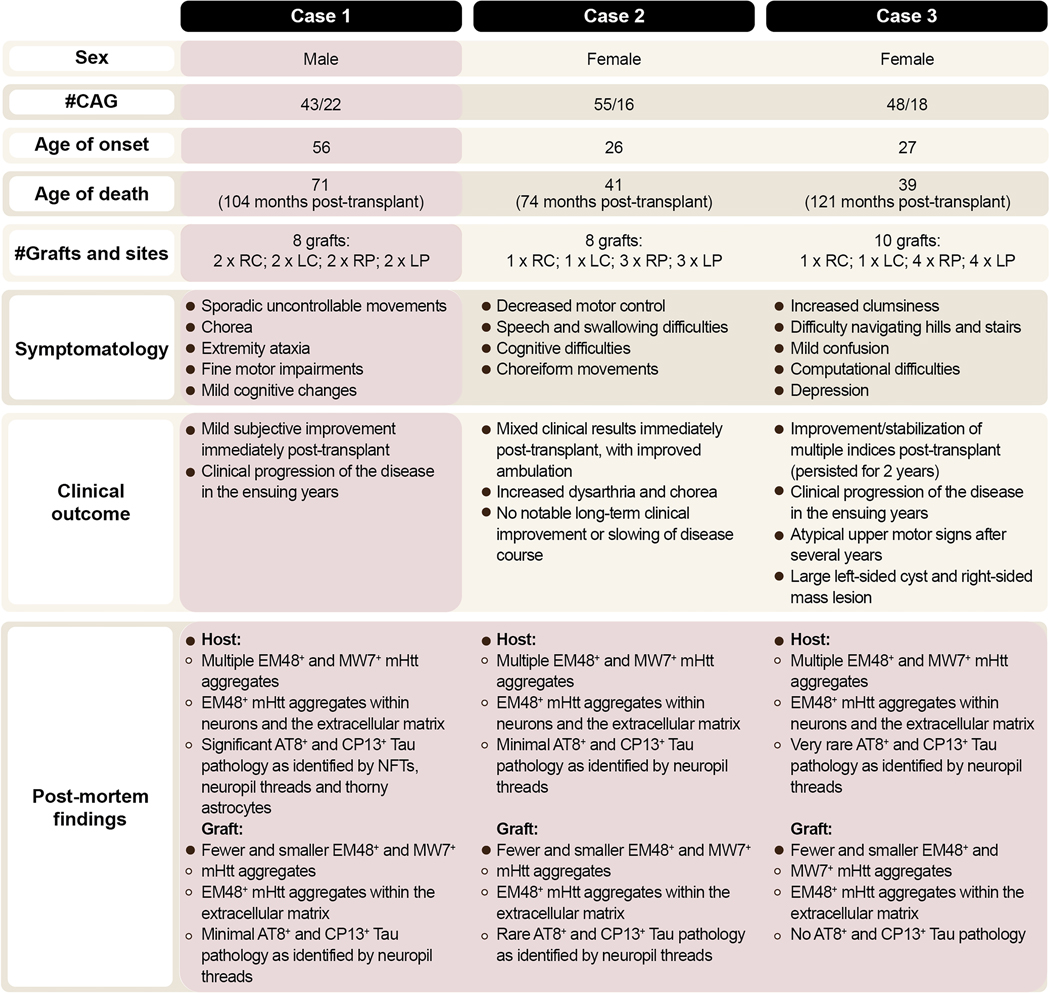
Summary of patients’ characteristics. Information of all three transplanted HD patients. Light pink boxes represent new details and observations not published previously. Abbreviations: mHtt = mutant huntingtin; NFTs = neurofibrillary tangles; LC = left caudate; LP = left putamen; Ref = reference; RC = right caudate; RP = right putamen; # = number.

All three patients had pathological CAG trinucleotide expansion in the *Htt* gene, MRI confirmation of striatal atrophy, striatal hypometabolism as measured by 2-deoxyglucose PET and chorea as one of the main clinical features (Kopyov et al., 1998) (Fig 1). All cases were evaluated using the Unified Huntington’s Disease Rating Scale (UHDRS) 3–10 weeks prior to surgery, as well as 6 and 12 months following transplantation for signs of improvements (Kopyov et al., 1998; Keene et al., 2009; Keene et al., 2007).

### 3.1. Clinical history

#### 3.1.1. Case 1

The subject was a 71-year-old man without a significant medical history until he began noticing sporadic uncontrollable jerking motions of his shoulders and arms in his mid to late forties. This progressed to insuppressible chorea, fine motor impairment, and mild cognitive changes. Genetic testing ultimately revealed 43 CAG repeats in the *Htt* gene locus. Family history was significant for a diagnosis of HD in his mother and maternal uncle, as well as two of his seven siblings. At age 63, he received bilateral fetal neural transplants, seven years post-diagnosis. He underwent placement of two grafts in each putamen and two grafts in each caudate nucleus (8 grafts in total). Approximately one month after the procedure he fell, leading to bilateral subdural hematomas that required evacuation. Six months post-transplantation, he reported feeling less fatigued and increased in weight from 126 to 137 lbs. However, he continued to experience balance issues and falls of increasing severity, with multiple resulting in loss of consciousness. By five years post-transplantation, he was exhibiting marked exaggerated chorea involving all extremities and trunk, dysarthria, depression, weight loss, and inability to ambulate without assistance. MRI reportedly identified severe global atrophy of the cortex with focal bilateral caudate atrophy. He died at age 71, 8 years post-transplantation (Fig 1).

#### 3.1.2. Cases 2 and 3

These cases have been previously reported (Keene et al., 2009; Keene et al., 2007) and the pertinent features are highlighted in Fig 1. Briefly, Case 2 was a 41-year-old female with HD who, at age 35, received a total of eight grafts bilaterally, which included three grafts per putamen and one graft per caudate nucleus (Keene et al., 2007). She died six years post-transplantation. Case 3 was a 39-year-old female with HD who underwent placement of four grafts in each putamen and one graft in each caudate nucleus (10 grafts in total) (Keene et al., 2009) at age 29. She died ten years post-transplantation.

### 3.2. Neuropathological examination

#### 3.2.1. Case 1

The whole brain weighed 1,140 grams and there was mild to moderate atrophy of the frontal lobes bilaterally. The cerebellum and brainstem were grossly unremarkable (Fig S1A, B). Four defects, measuring up to 2 mm each, were identifiable in the bilateral middle frontal gyri. There was rust discoloration of the tissue surrounding these anomalies but there was no evidence of acute hemorrhage (Fig S1C). There was moderate atherosclerosis and the posterior communicating arteries were abnormally small. Upon coronal sectioning, there was no evidence of edema, herniations, or mass lesions. The caudate nuclei were severely atrophic, with a concave appearance, and the lateral ventricles were moderately enlarged (Fig S1D, E). Grossly, these features are consistent with a Vonsattel grade 4 of 4 (Vonsattel et al., 1985). The hippocampi were mildly atrophic. No infarcts or hemorrhages were identified. Four distinct nodules, measuring 0.5 to 1.8 cm in greatest dimension, were -observable in each of the right and left neostriata, with one nodule each in the anterior caudate, middle caudate, anterior putamen, and posterior putamen, bilaterally. The nodule in the left middle caudate projected into the adjacent internal capsule (Fig S1E, F).

Histologically, sections of bilateral caudate and putamen showed severe loss of neurons and marked astrogliosis extending from anterior to posterior. Overall, the histological features were consistent with a Vonsattel grade 4 of 4. Htt-positive intranuclear and intracytoplasmic neuronal inclusions, as well as occasional extracellular inclusions, were readily identified in the host caudate nucleus and putamen as well as in sections of frontal cortex, which also demonstrated mild gliosis. Other pathological findings included focal neurofibrillary tangles in the transentorhinal cortex, consistent with a Braak stage I, as well as patchy, perivascular thorny astrocytes containing the abnormal tau protein. Other tau pathology included occasional threads within neocortex. On standard hematoxylin and eosin stains, the grafts consisted of neuronal and glial elements with a variably gliotic graft-host border.

#### 3.2.2. Cases 2 and 3

Previous publications of these cases also reported on the neuropathological findings (see (Keene et al., 2009; Keene et al., 2007) for details). Briefly, for case 2, the brain weighed 1,145 g and had mild to moderate frontal cortical atrophy, severe ventricular dilatation, and severe atrophy of the caudate and putamen bilaterally. Microscopically, the striatum showed marked astrogliosis with severe neuron loss and common neuronal intranuclear ubiquitin+ immunoreactive inclusions. The grafts were well-circumscribed and associated with a tract of intense astrogliosis interpreted as a scar secondary to cannulation. Away from needle tract gliosis, the borders between host and graft tissue did not display significantly increased astrocytic reaction. For case 3, the brain weighed 985 g with mild frontal cortical atrophy and severe atrophy of the caudate nuclei. Seven cannula tracts were identified in the middle frontal gyri and coronal sections revealed multiple well-circumscribed grafts in the bilateral caudate and putamen, some of which showed overgrowth and cystic change. Histological examination revealed intense gliosis of the bilateral caudate nuclei and putamen. Examination of the grafts uncovered circumscribed masses composed of unorganized neuropil with a population of mostly small and some large neurons interspersed with islands of reactive astrocytes and scattered oligodendrocytes. The masses also contained individual and bundled myelinated axons that rarely crossed the graft-host boundary.

### 3.3. Detection of mHtt aggregates and forms of phosphorylated tau within the grafted tissue

Additional post-mortem analyses were carried out to specifically investigate the presence of mHtt aggregates and tau pathology within the grafted tissue from all three cases. Double immunohistochemical staining was performed to detect mHtt aggregates and hyperphosphorylated tau in the grafted tissue using two different antibodies for each analyte.

#### 3.3.1. Case 1

**Fig. 2.**
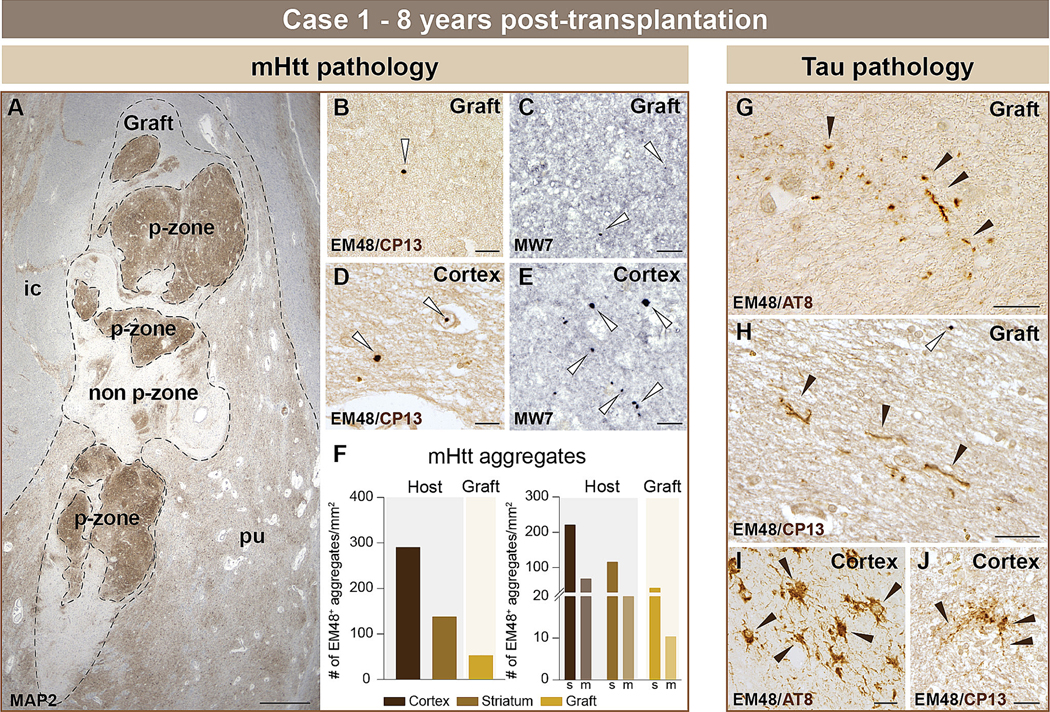
Histological evaluation of mHtt and phosphorylated tau within the host cortex and neuronal fetal allografts 8 years post-transplantation (case 1). Low power magnification image of one fetal tissue graft located in the putamen (A). Areas rich in striatal cells, referred to as p-zones, were further outlined (dotted line). Double immunohistochemical labelling revealed mHtt aggregates (Ni-DAB, dark purple to black) in the grafted tissue using both EM48 (B) and MW7 (C) antibodies (white arrows). As expected, mHtt aggregates (Ni-DAB, dark purple to black) were also detected in the host cortex using either EM48 (D) or MW7 (E) antibodies (white arrows) and quantifications were conducted in the host cortex, striatum and grafted tissue (F). Aggregates were further counted by volume size of either small (<0.75μm^3^) or medium/large (≥0.75μm^3^) (F). Phosphorylated tau (DAB brown) neuropil threads were detected in the graft using either AT8 (G) (phospho-tau Ser202 and Thr205) or CP13 (H) (pSer202) antibodies (black arrows). Phosphorylated tau (DAB brown) tangles and neuropil threads were observed in the host cortical tissue with AT8 (I) and CP13 (J) antibodies (black arrows). Scale bars: A = 1 cm; B-E, G-J = 20 μm. Abbreviations: ic = internal capsule; m = medium; MAP2 = microtubule-associated protein 2; mHtt = mutant huntingtin; pu = putamen; s = small; # = number.

We studied the graft located within the host putamen (Fig 2A). A few small (ranging mostly from 0.1 – 5 μm) EM48^+^ mHtt aggregates were present within the grafted tissue (Fig 2B, white arrows); an observation which we validated using anti-mHtt MW7 antibody (Fig 2C, white arrows). EM48^+^ mHtt aggregates of various sizes (ranging from 0.1 – 15 μm) were found in the host cortical and striatal tissue, as well as in the vicinity of the graft (Fig 2D, white arrows). These aggregates were also identified using the MW7 antibody (Fig 2E, white arrows). Quantification of the total number of EM48^+^ mHtt aggregates revealed much greater numbers within the host cortex and striatum than within the graft (Fig 2F). In addition to mHtt, the grafted tissue was characterized by tau pathology which was restricted to rare tau^+^ neuropil threads, as detected using both AT8 (phospho-tau Ser202 and Thr205) (Fig 2G, black arrows) and CP13 (pSer202) (Fig 2H, black arrows). In the host cortex, several tau immunoreactive elements (neurofibrillary tangles (NFTs), neuropil threads and thorny astrocytes) were variably present using both AT8 (Fig 2I, black arrows) and CP13 (Fig 2J, black arrows) antibodies.

#### 3.3.2. Case 2

**Fig. 3.**
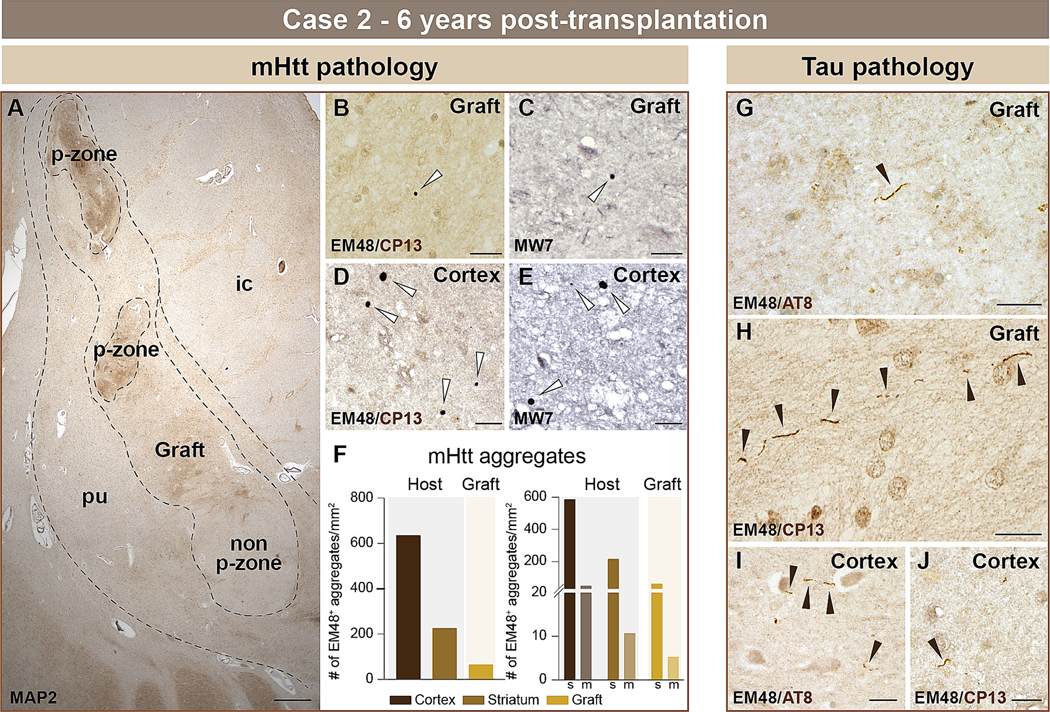
Histological evaluation of mHtt and phosphorylated tau within the host cortex and neuronal fetal allografts 6 years post-transplantation (case 2). Low power magnification image of one fetal tissue graft located in the putamen (A). Areas rich in striatal cells, referred to as p-zones, were further outlined (dotted line). Double immunohistochemical labelling revealed mHtt aggregates (Ni-DAB, dark purple to black) in the grafted tissue using both EM48 (B) and MW7 (C) antibodies (white arrows). As expected, mHtt aggregates (Ni-DAB, dark purple to black) were also detected in the host cortex using either EM48 (D) or MW7 (E) antibodies (white arrows) and quantifications were conducted in the host cortex, striatum and grafted tissue (F). Aggregates were further counted by volume size of either small (<0.75μm^3^) or medium/large (≥0.75μm^3^) (F). Phosphorylated tau (DAB brown) neuropil threads were detected in the graft using either AT8 (G) (phospho-tau Ser202 and Thr205) or CP13 (H) (pSer202) antibodies (black arrows). Phosphorylated tau (DAB brown) neuropil threads were observed in the host cortical tissue with AT8 (I) and CP13 (J) antibodies (black arrows). Scale bars: A = 1 cm; B-E, G-J = 20 μm. Abbreviations: ic = internal capsule; m = medium; MAP2 = microtubule-associated protein 2; mHtt = mutant huntingtin; pu = putamen; s = small; # = number.

We investigated the graft located within the host putamen (Fig 3A). mHtt aggregates were found in the grafted tissue using both EM48 (Fig 3B, white arrows) and MW7 (Fig 3C, white arrows) labelling and were identified in the host cortex using the same antibodies (Fig 3D, E, white arrows). EM48^+^ aggregates found in the grafted tissue were smaller (ranging from 0.1 – 5 μm) than the cortical and striatal aggregates (ranging from 0.1 – 15 μm) and were much less numerous (Fig 3F). Only a few AT8^+^ and CP13^+^ tau neuropil threads were noticeable in the grafted area and the occurrence of such tau^+^ threads was much sparser than observed in case 1 (Fig 3G, H, black arrows). Similarly, AT8^+^ and CP13^+^ neuropil threads were rarely identified in the host cortex (Fig 3I, J, black arrows). Overall, the extent of tau pathology was mild and no NFTs were found in either the host or the grafted tissue.

#### 3.3.3. Case 3

**Fig. 4.**
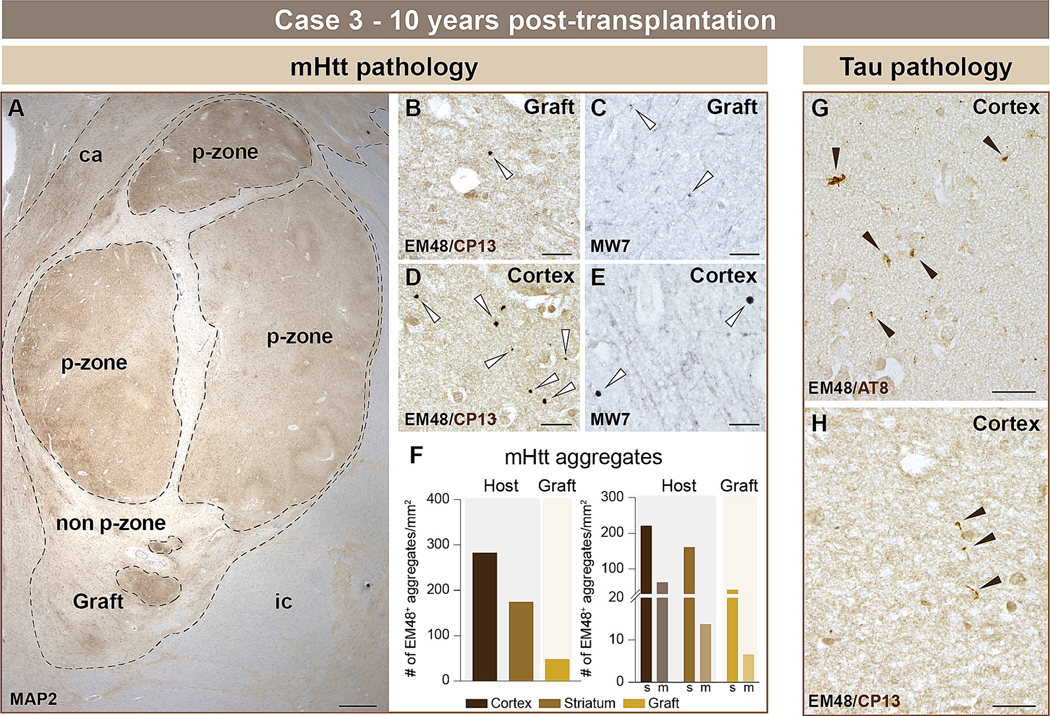
Histological evaluation of mHtt and phosphorylated tau within host cortex and neuronal fetal allografts 10 years post-transplantation (case 3). Low power magnification image of one fetal tissue graft located in the caudate (A). Areas rich in striatal cells, referred to as p-zones, were further outlined (dotted line). Double immunohistochemical labelling revealed mHtt aggregates (Ni-DAB, dark purple to black) in the grafted tissue using both EM48 (B) and MW7 (C) antibodies (white arrows). As expected, mHtt aggregates (Ni-DAB, dark purple to black) were also detected in the host cortex using either EM48 (D) or MW7 (E) antibodies (white arrows) and quantifications were conducted in the host cortex, striatum and grafted tissue (F). Aggregates were further counted by volume size of either small (<0.75μm^3^) or medium/large (≥0.75μm^3^) (F). Phosphorylated tau (DAB brown) neuropil threads were observed in the host cortical tissue using either AT8 (G) (phospho-tau Ser202 and Thr205) or CP13 (H) (pSer202) antibodies (black arrows). Scale bars: A = 1 cm; B-E, G-H = 20 μm. Abbreviations: ca = caudate; ic = internal capsule; m = medium; MAP2 = microtubule-associated protein 2; mHtt = mutant huntingtin; s = small; # = number.

We analyzed the graft located in the host caudate nucleus (Fig 4A). Sparse EM48^+^ (Fig 4B, white arrows) and MW7^+^ (Fig 4C, white arrows) aggregates were observed within the grafted tissue. mHtt aggregates (ranging from 0.1 – 15 μm) were detected with both antibodies in the host tissue (Fig 4D, E, white arrows) and were widely distributed throughout the host cortex and striatum (Fig 4F). While no signs of tau pathology were observed in the graft using two different anti-tau antibodies (AT8 and CP13), rare AT8^+^ (Fig 4G, black arrows) and CP13 (Fig 4H, black arrows) neuropil threads were identified in the host cortex. This case showed the least tau immunoreactivity in the host tissue amongst the three transplanted HD cases investigated in this study.

### 3.4. Localization of EM48+ mHtt aggregates in the host and grafts

**Fig. 5.**
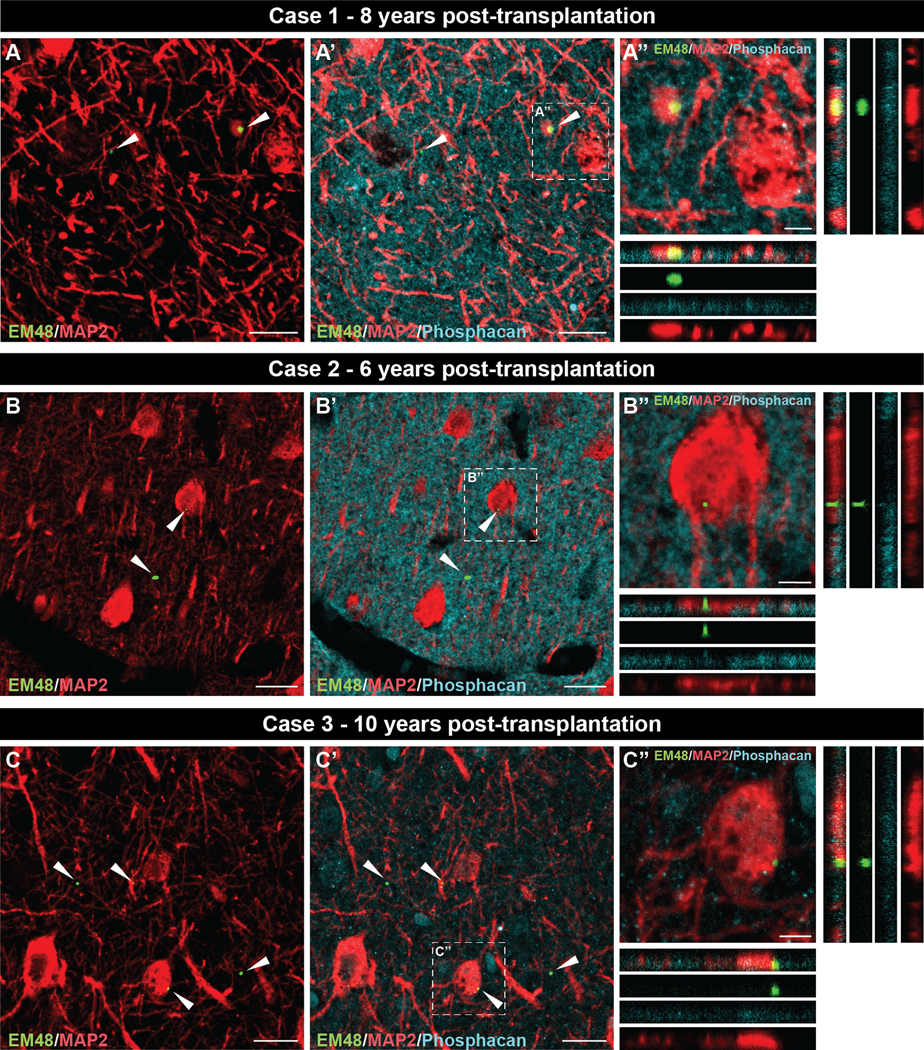
Localization of EM48^+^ mHtt aggregates in the host cortical tissue of all three cases. Triple immunofluorescence labelling of EM48 (mHtt aggregates, green; white arrows), MAP2 (neuronal marker, red) and phosphacan (extracellular matrix, cyan) in HD host cortex of case 1 (A, A’), case 2 (B, B’) and case 3 (C, C’). Orthogonal views, from the selected area delineated with a dotted line in (A’, B’, C’), enabled the visualization of EM48^+^ mHtt aggregates within the extracellular matrix (phosphacan) as well as within neuronal elements (MAP2) (A”, B”, C”). Scale bars: A, A’, B, B’, C, C’ = 20 μm; A”, B”, C” = 5 μm. Abbreviations: MAP2 = microtubule-associated protein 2; mHtt = mutant huntingtin.

**Fig. 6.**
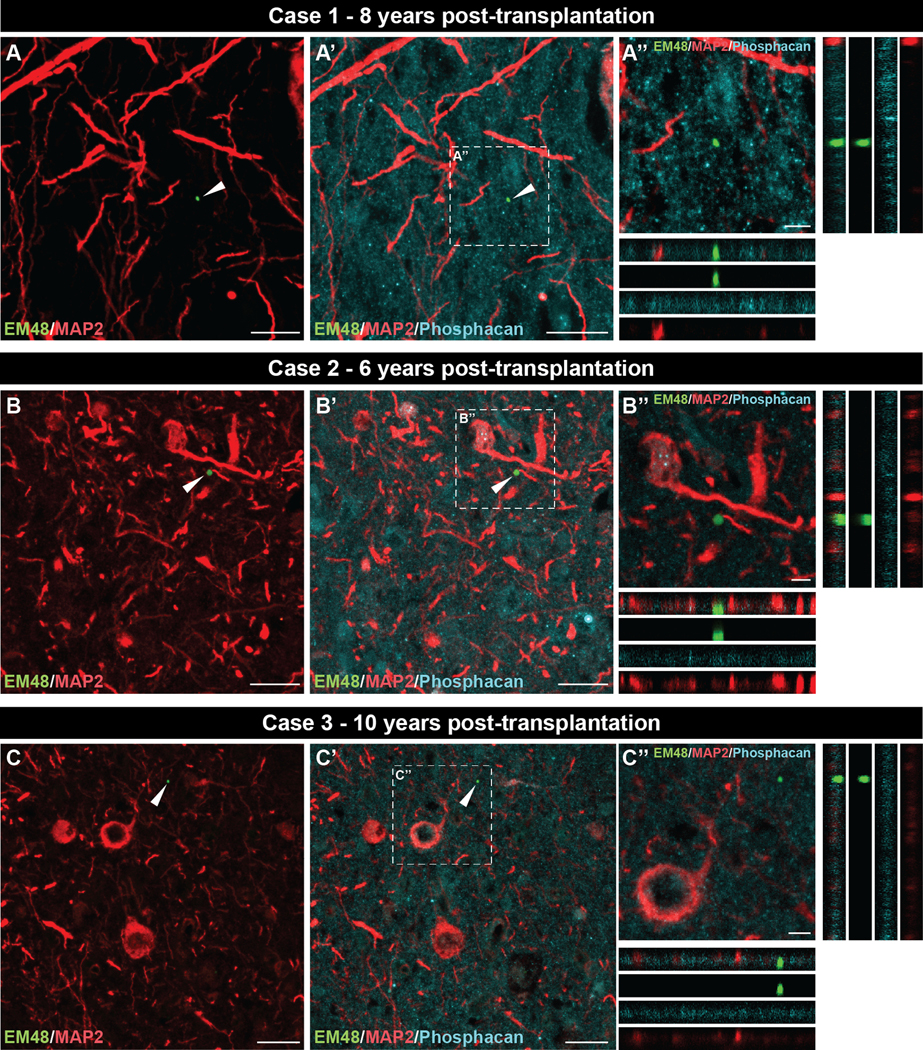
Localization of EM48^+^ mHtt aggregates in the grafted tissue of all three cases. Triple immunofluorescence labelling of EM48 (mHtt aggregates, green; white arrows), MAP2 (neuronal marker, red) and phosphacan (extracellular matrix, cyan) in the grafted tissue of case 1 (A, A’), case 2 (B, B’) and case 3 (C, C’). Orthogonal views, from the selected area delineated with a dotted line in (A’, B’, C’), enabled the visualization of EM48^+^ mHtt aggregates exclusively within the extracellular matrix (phosphacan), and not with the neuronal marker MAP2 (A”, B”, C”). Scale bars: A, A’, B, B’, C, C’ = 20 μm; A”, B”, C” = 5 μm. Abbreviations: MAP2 =, microtubule-associated protein 2; mHtt = mutant huntingtin.

The localization of EM48^+^ mHtt aggregates within or outside neuronal elements (i.e., extracellular matrix) was evaluated in both the host (Fig 5) and the grafted tissue (Fig 6) by triple immunofluorescence staining. For all three cases, aggregates present in the host cortex were detected in both the extracellular matrix (phosphacan) and within neurons (MAP2) (Fig 5) as orthogonal views confirmed EM48/MAP2 colocalization (Fig 5A”, B”, C”). Analysis of all three transplants (Fig 6) revealed the presence of smaller EM48^+^ deposits in the extracellular matrix but not within neurons as we failed to observe colocalization with MAP2 staining (Fig 6A”, B”, C”).

## 4. Discussion

The primary objective of this study was to utilize the post-mortem brain tissue available from several transplanted HD patients in the Los Angeles trial to validate prior observations that mHtt and hyperphosphorylated tau can be detected in genetically unrelated grafted tissue in HD patients (Cicchetti et al., 2014; Cisbani et al., 2017). In the three transplanted HD cases presented here, small but rare mHtt aggregates were found in the grafts, while mHtt aggregates of various sizes were widely distributed in the host striatum and cortex. Focal tau pathology was also observable in the host tissue of all three cases while variable tau neuropil threads were visible within the grafts in two out of the three investigated cases.

The current observation that aggregated mHtt is present within genetically unrelated solid grafts is in line with our previous report on another post-mortem study of transplanted HD patients where mHtt aggregates were consistently detected in solid tissue grafts of three cases who came to autopsy more than a decade post-transplantation (Cicchetti et al., 2014). These findings were also previously confirmed in an additional HD case from NEST-UK multicenter trial where patients received cell suspension allografts instead of solid tissue grafts (Maxan et al., 2018). Previous reports from Keene et al (Keene et al., 2009; Keene et al., 2007) did not find mHtt inclusions in the grafted tissue but only in the host. The discrepancy in these observations can be due to a number of reasons including variations in tissue sampling and immunohistochemical protocols used to locate mHtt. In the current study, we validated previous findings using two different immunohistochemical paradigms employing an anti-mHtt EM48 antibody, which specifically targets the aggregated mHtt protein (epitope: amino acid 1–256), and a MW7 antibody that binds to the PolyP (proline) rich region of mHtt. As part of this validation process, EM48 immunostaining was carried out on multiple sections, from all three cases and in two independent laboratories.

We have also investigated the location of mHtt aggregates at the cellular level and found that they were not detected within the grafted neurons, but were niched within the extracellular matrix. In the host cortex, however, aggregates were detected within both neuronal elements and the extracellular matrix. This is in line with a previous study in which we have reported the mHtt aggregates within the graft were mainly present in the extracellular matrix and colocalized with matrix proteins such as phosphacan (Cicchetti et al., 2014). Thus, the current study is a strong validation of our previous observations regarding the presence of mHtt aggregates within the extracellular matrix of the grafted tissue.

In this report, we also replicate previously published findings on the presence of hyperphosphorylated tau in the HD brain (Vuono et al., 2015; St-Amour et al., 2018; Petry et al., 2023) as well as within allografted tissue in patients with HD (Cisbani et al., 2017). However, there appears to be an association of the tau pathology with age; the 71-year-old subject had significantly more tau pathology than either of the younger subjects, and pattern of tauopathy present in the older subject was most consistent with previously described age-related tau pathology often observed at autopsy in aging cohorts. Specifically, the neuronal tau pathology followed the pattern of primary age-related tauopathy and the glial tau fits descriptions of age-related tau astrogliopathy (Crary et al., 2014; Kovacs et al., 2016). Alternatively, or in combination, Htt may play a role in the initiation or progression of tauopathy. Indeed, we observed that all three HD transplanted cases exhibited various degrees of tau pathology in different structures of the cerebral tissue (i.e. cortex and striatum), as previously reported (Cisbani et al., 2017; Vuono et al., 2015; Fernández-Nogales et al., 2014; Gratuze et al., 2016; Salem and Cicchetti, 2023). We also found rare tau^+^ neuropil threads in the grafted tissue of the 71- and 41-year-old transplanted HD patients 8 and 6 years following surgery, respectively. Of note, tau immunolabelling was not seen in the host striatum, nor in the grafted tissue of the 39-year-old case, and only a few tau^+^ threads were visible in cortical areas. Tau pathology is a common feature of many neurodegenerative processes, including those related to chronic repetitive neurotrauma and those associated with aging. Therefore, it is uncertain if the tau observed is related to aging, the surgical trauma of the graft placements, head injury from repeated falls, or the underlying HD pathology. On a larger scale, our results hint at a role of tau in HD pathology, which has, in recent years, been increasingly investigated (Blum et al., 2015; Gratuze et al., 2015; L’Episcopo et al., 2016; Liu et al., 2019; Alpaugh et al., 2022; Mees et al., 2022a; Petrozziello et al., 2023). Despite discrepencies in some animal experiments (Mees et al., 2022b), results do convergence towards a contribution of tau to various HD-related features (Salem and Cicchetti, 2023).

## 5. Conclusions

Our observations indicate that pathogenic mHtt and tau protein can be found in healthy fetal tissue years following transplantation, however, the inherent limitations of post-mortem analysis of brain tissue prevent us from drawing firm conclusions on the possibility of cell-to cell or region-specific propagation of mHtt from the host to the transplanted tissue. While additional investigations are required to decipher the mechanisms of pathological protein spread in the human brain, a few possibilities can be considered: (i) mHtt may have been transported from cortical projections which connect to striatal neurons, and in particular grafted striatal neurons (Freeman et al., 2000b). (ii) mHtt in the graft may be a residual debris of the degeneration of cortical or striatal neurons containing mHtt. This hypothesis can be supported by our observations reported in Cicchetti et al (Cicchetti et al., 2014) and validated in this study, where mHtt aggregates did not colocalize with MAP2^+^ grafted neurons but were found proximal to these neuronal elements. (iii) Localization of mHtt in the graft can be due to diffusion of mHtt protein from the host striatum to the transplant via the extracellular matrix. This possibility is based on studies demonstrating dissemination of soluble amyloid-β through the extracellular space and its uptake by cells in near vicinity (Meyer-Luehmann et al., 2003; Soto, 2012). (iv) Of significant importance, studies in mice have demonstrated prion-like transmission of mHtt aggregates to genetically unrelated healthy tissue resulting in marked behavioral and pathological HD-related phenotypes (Jeon et al., 2016; Masnata et al., 2019; Srinageshwar et al., 2020).

Taken together, we have now collected data from three individual trials in which we consistently identified both mHtt within the grafts accompanied by mild tau pathology in cases more recently investigated. The clinical implications of such findings remain to be elucidated.

## Supplementary Material

Supplemental figure 1

Supplementary data to this article can be found online at https://doi.org/10.1016/j.nbd.2024.106542.

## Data Availability

Data will be made available on request.
