# Supplementary figures and images for: Evidence of mutant huntingtin and tau-related pathology within neuronal grafts in Huntington’s disease cases
Source: Neurobiol Dis. Author manuscript; Available in PMC 2025 Sep 19. (PMC12448652; doi:10.1016/j.nbd.2024.106542)

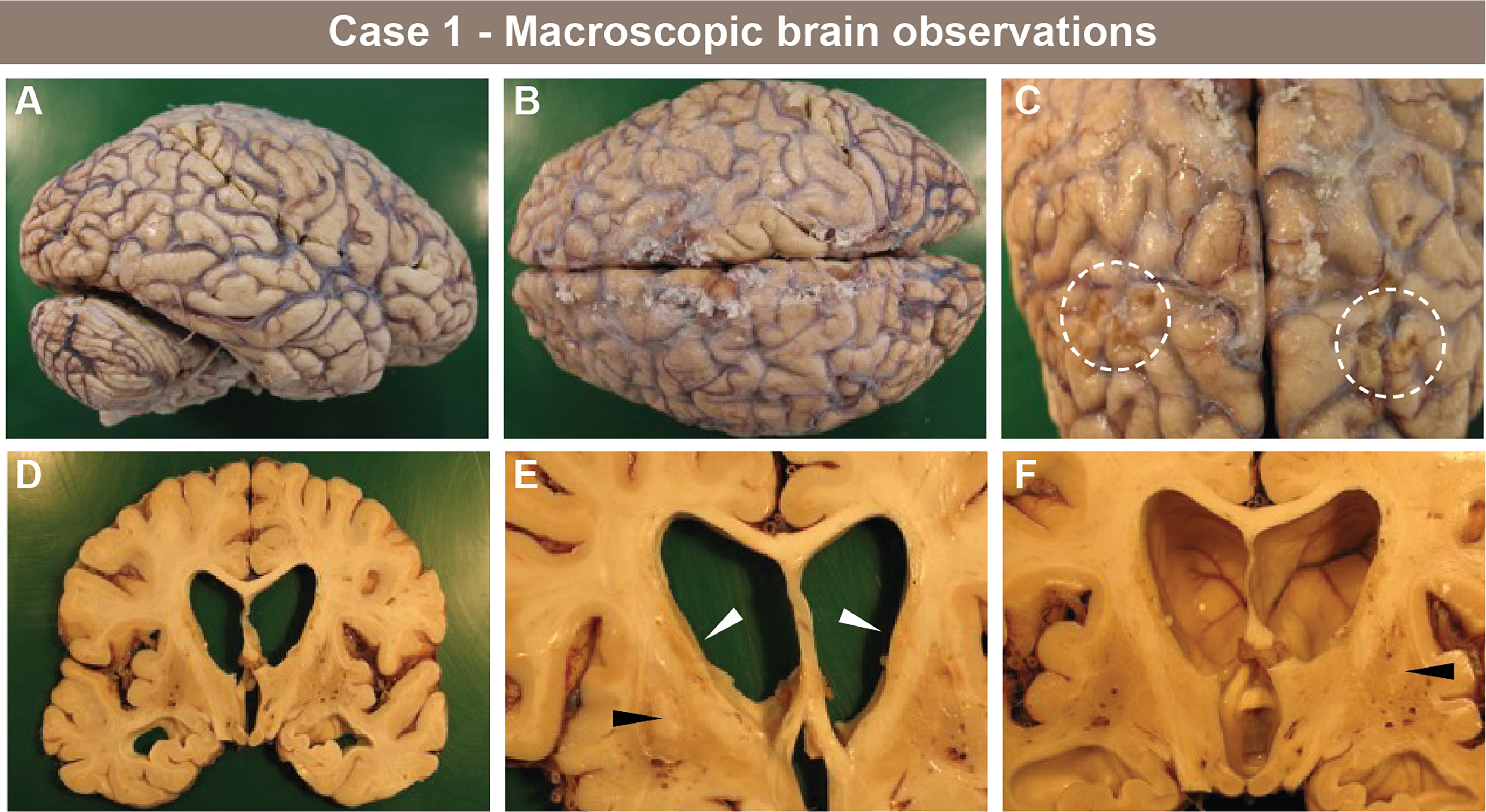

Supplement: Supplemental figure 1 [file NIHMS2064307-supplement-Supplemental_figure_1.jpg]
